# What Do Arithmetic Errors in the Financial Context Reveal? A Preliminary Study of Individuals with Neurocognitive Disorders

**DOI:** 10.3390/neurolint15020046

**Published:** 2023-06-01

**Authors:** Vaitsa Giannouli, Magdalini Tsolaki

**Affiliations:** School of Medicine, Aristotle University of Thessaloniki, 541 24 Thessaloniki, Greece

**Keywords:** arithmetic errors, types, MCI, AD, PDD, healthy aging

## Abstract

Objectives: Arithmetic errors in the financial context have been investigated mainly in cognitively normal Parkinson’s disease (PD) patients and mildly impaired PD (PD-MCI) individuals. The aim of this study was to examine arithmetic errors in the financial context across neurocognitive disorders. Methods: Four hundred and twenty older adults from Greece were divided into four groups (110 patients with a diagnosis of Alzheimer’s disease (AD), 107 patients with a diagnosis of mild cognitive impairment (MCI), 109 healthy controls and 94 Parkinson’s disease dementia (PDD) patients). Their ages ranged from 65 to 98 years (M = 73.96, SD = 6.68), and the sample had a mean of 8.67 (SD = 4.08) years of education. For each of the AD patients, a counterpart matched by age, educational attainment and gender was selected from a larger group of participants. Results: Overall, the results reveal that healthy older adults did not commit arithmetic errors, but AD patients reported procedural errors in their responses to both questions. A high frequency of procedural errors was found in MCI patients’ responses to the first question, while the errors in their responses to the second question cannot be categorized. Finally, in PDD patients, place value errors were reported for the first question, while more magnitude errors were made when responding to the second question. Conclusions: These findings support that arithmetic errors within financial contexts are not the same across neurocognitive disorders, and numerical representations are not impaired not only in PDD, but also in AD and MCI. This information could be useful in cognitive assessments performed by neurologists and neuropsychologists as these types of errors may be indicators of specific brain pathologies.

## 1. Introduction

It is widely accepted that arithmetic functioning is negatively influenced by neurocognitive disorders and their severity [1,2,3]. Specific brain areas such as the left angular gyrus and amygdala have been found to be involved in financial capacity tasks and dyscalculia even in mildly affected patients, such as those with a diagnosis of mild cognitive impairment (MCI) [4,5].

For example, the predictive role of specific arithmetic tasks, such as one item from the Mini Mental State Examination (MMSE) that calls for the serial subtraction of serial sevens, has been found to have a very powerful tool for determining financial capacity in neurocognitive disorders [6], therefore rendering it a useful tool in clinical practice. Of course, arithmetic errors are generally expected in older patients (even those without brain pathologies [7]), and there are findings supporting specific perseveration errors that can also be found in healthy adults performing mathematic tasks [8].

The importance of arithmetic errors in relation to instrumental activities of daily living, as well as legal and financial issues, in older age has been demonstrated in the older Greek population with the Legal Capacity for Property Law Transactions Assessment Scale (LCPLTAS). This tool includes a section specifically focusing on arithmetic abilities. Several studies support overall deficits in arithmetic capacities and financial capacities across a diverse range of disorders, such as vascular dementia, Alzheimer’s disease (different stages and severity) and Parkinson’s disease [9]. An overemphasis is currently placed on nonmotor symptoms (i.e., hyposmia, depression and sleep behavior disorders) associated with PDD development [10]. Additionally, cognitive deficits regarding arithmetic/financial cognition have been reported in amnestic MCI (aMCI) patients [9], frontotemporal dementia (FTD) patients [9] and Lewy body dementia patients [9]. Although there is evidence of arithmetic deficits in all of these disorders [9], to date no systematic research has investigated the exact nature of the errors.

According to the grouping proposed by Nuerk et al. [11] and discussed in detail by Loenneker et al. [12], arithmetic errors using Arabic numbers, although heterogeneous, can be grouped in three ways: erroneous magnitude processing, which shows up as rounding errors within the correct decade (i.e., 3 × 6 = 16, not 18); impaired calculation procedures, which can arise as operand (i.e., 3 × 6 = 12) or operation errors (i.e., 3 × 6 solved as 3 + 6); and errors regarding decade value (i.e., 3 × 6 = 28), place value (3 × 6 = 180) or both (3 × 6 = 280), which stress an impaired place value integration.

The aims of this study were twofold. The research team planned to (1) examine whether there are specific types of arithmetic errors found in specific diagnoses of neurocognitive disorders; and (2) investigate if findings from research in different cultural contexts can also be applied in Greece.

## 2. Method

Four hundred and twenty older adults (228 women, 192 men) with a diagnosis of neurocognitive disorder as well as healthy older adults were recruited from a larger pool of participants. Participants were residents of Thessaloniki and other areas in Northern Greece. Participants completed a demographics questionnaire and underwent a brief neuropsychological assessment (separate from the detailed examination to confirm their diagnosis). Participant examinations included the Mini Mental State Examination (MMSE) [13] and the Geriatric Depression Scale (GDS-15). Scores from the MMSE and GDS were used to exclude participants with comorbid depressive symptomatology [14]. In the case of participants who were unable to complete the forms, caregiver assistance was utilized to gather relevant information. The examiner was blind to the participants’ diagnosis. The diagnoses were made prior to the neuropsychological assessments and were valid during the assessments. Most of the patients had not started drug therapy at the time of assessment.

The inclusion criteria for the three groups of patients were as follows: (a) a diagnosis of MCI based on the criteria of the *Diagnostic and Statistical Manual of Mental Disorders*, *Fifth Edition* (DSM-5); (b) a diagnosis of AD based on whether the patients’ profile followed the criteria of DSM-5 for major neurocognitive disorder due to AD, supported by neuroimaging (MRI) and computed tomography (CT) testing, and confirmation of relevant biomarkers used at the G. Papanikolaou Hospital; (c) a diagnosis of PDD, based on the criteria in DSM-5 for major neurocognitive disorder due to PD. Exclusion criteria for the community-dwelling group of healthy controls were a history/diagnosis of MCI or other type of neurocognitive disorder, functional cognitive disorders, diagnosis of mental, psychiatric and/or other neurological disorder (e.g., hydrocephalus), history of traumatic brain injury (TBI), history of a serious physical disease/illness (e.g., cancer, thyroid disorders, diabetes, heart disease), substance/alcohol abuse and uncorrected sensory deficits (visual and auditory). For all groups, inclusion criteria included (a) being above 65 years of age; (b) having scores below 6/15 on the GDS for the exclusion of depressive symptomatology; and (c) being native speakers of Greek.

All participants were recruited from the Memory and Dementia Outpatient Clinic of the 3rd University Department of Neurology, “G. Papanikolaou” General Hospital, in Northern Greece. Community-dwelling participants were recruited via flyers and oral announcements at local senior centers. We used convenience sampling for this group of participants, so we could match individuals based on the demographic characteristics of the patient groups. The study was approved by the ethics committee (Aristotle University of Thessaloniki: Decision 2, 27 March 2013) following the Declaration of Helsinki. The assessments took approximately 12 months to perform. Written informed consent was acquired. Participants did not obtain financial or other forms of compensation for their voluntary participation.

Two financial arithmetic questions from the Clinical Dementia Rating interview (CDR; [15]) were presented orally: “How many 5 cent coins make up 1 €?” and “How many 50 cent coins make up 15.50 €?” The decision to present these questions orally instead of in written form was because individuals must use mental arithmetic in everyday life in a plethora of similar situations. These two tasks have face and construct validity. The constructs being measured are the everyday financial tasks individuals are required to perform. The two questions also demonstrate criterion validity and concurrent/convergent validity as other neuropsychological tests focusing exclusively on financial capacity include similar arithmetic capacity items (e.g., [9]). Participants answered in a verbal open-response format and were recorded by the researcher. These responses were assessed based on correctness and errors where present.

Errors were ascribed to distinct categories as in previously published research [12]. Specifically, for these two questions, the grouping of arithmetic errors presented in Loenneker et al. [12] was applied. More specifically, for the category of place value error, wrong decade value (i.e., 10 for the first question and 11 for the second), wrong place (2 or 200) and wrong place and decade (100) were included. The magnitude error category refers to rounding with correct decade (30 or 32). The procedural error category includes operant errors (with solution in multiplication table) (i.e., 15), wrong operations (for example, division and not multiplication (7.75) and wrong operation and place value integration (76 = 15.50 ÷ 2 × 10). Other errors include arbitrary (4 for the first question and 4 or 8 or 23 or 25 or 26 for the second) and/or repetition of operand (15). The NA error category (no information or exact error is given as a response) used in [12] was not applied as all participants were prompted to give a response, even if it was an incorrect one.

## 3. Results

Participants were matched on biological sex (χ^2^(3) = 5.331, *p* = 0.149), age (F(3, 416) = 2.547, *p* = 0.056) and years of education (F(3, 410) = 2.348, *p* = 0.072) (Table 1). Regarding their socioeconomic status, all participants came from a middle-class background, as measured by yearly income.

The chi-square test along with adjusted standardized residuals was used for detecting differences in the types of responses among the four diagnostic groups for the two questions. A chi-square test of independence revealed a statistically significant association between diagnosis and type of error response for the first question (χ^2^(12) = 355.778, *p* < 0.001). More specifically, adjusted residuals that were more than 1.96 (2.0 was used by convention) indicated the number of cases in that cell was significantly larger than would be expected if the null hypothesis (no difference) were true, with a significance level of 0.05. In this line, adjusted residuals that were less than −2.0 indicated that the number of cases in that cell was significantly smaller than would be expected if the null hypothesis were true (Table 2).

For the second question, a similar significant association between diagnosis and type of error response was found (χ^2^(12) = 224.125, *p* < 0.001) (Table 3).

## 4. Discussion and Conclusions

These preliminary results demonstrate clinically relevant arithmetic errors in financial contexts. More specifically, the results corroborate the findings of a previous study focused on PD and PD with MCI [12], but not PDD. Thus, although there is an overlap with findings from older German adults with a diagnosis of PD, this study shows for the first time that compared to other groups, there seems to be a pattern of arithmetic mistakes. As expected, healthy older adults did not commit arithmetic errors in general, although there is research supporting an age-related decline in an individual’s capacity to solve simple and complex arithmetic tasks involving processing speed (reaction time) and correctness of response, due to the normal aging process affecting working memory and executive skills [9,16,17,18,19,20,21,22,23]. This discrepancy may be due to the incomplete neuropsychological and physical assessment used in prior studies to ensure the inclusion of healthy older adults, as well as the tasks not mirroring those encountered in everyday life. In some previous studies, the assessments were also administered in a limited time frame, thus possibly causing high levels of anxiety in the participants. In the current study, AD patients made procedural and other unclassified errors when answering both questions. MCI patients made more procedural errors when responding to the first question and other unclassified errors for the second question. Finally, for PDD patients, place value errors followed by magnitude errors were reported most frequently for the first question and magnitude errors were most frequently made when responding to the second question. The above provides evidence that numerical representations are not the same across neurological impairments. These findings could be used in cognitive assessments as making these types of errors may be a sign of specific brain pathology in older adults.

Previous research supports the idea that calculation errors do occur as AD progresses, and calculation abilities tend to deteriorate hierarchically [24,25,26,27,28,29]. The skills for basic operations are the last to be affected by brain pathology, while more complex skills being lost first [30], although there may be some exceptions depending on the prior education and cognitive reserve of the individual [31,32]. It is interesting that although participants in this study did not have to deal with multiple-step arithmetic problems and the two administered tasks can be described as non-demanding, prominent deficits were observed for all groups, except for the healthy participants. For the PDD group, everyday activities involving numerical skills were affected, in contrast to prior findings that support difficulties only in performing formal numerical tasks (e.g., mental multiplication), and not everyday activities involving numbers [33]. Detection of such deficits should be included in assessment protocol testing.

All the above results have direct implications for clinical assessments for all groups of participants and especially for PDD patients. So far, both in research protocols and especially in clinical practice, the above points have been disregarded. Thus, arithmetic functions must be included in neuropsychological–cognitive assessments, and specifically designed instruments must be adopted and validated cross-culturally for this purpose. In this way, older individuals, as well as their family members and/or caregivers, can be informed and prepare for the possibility of financial abuse due to cognitive deficits causing vulnerability.

## 5. Limitations

The major limitations of this study were that (a) there were more women than men in the total sample and (b) the level of education in years in Greece is lower compared to that in other Western countries [26]. These two points should not be considered major drawbacks, since according to the Hellenic Statistical Authority, there are more older women (aged 65 and older) than men in general, and elders in Greece have had lower school attendance compared to other Western countries since World War II. The arithmetic errors under study involved only two oral mental calculation problems, and thus represent a very small and arguably unrepresentative sample of the broad range of patients’ everyday arithmetic errors. Therefore, concerns were raised about the diagnostic sensitivity of the error types across the examined groups of patients. Additionally, in this study, women were equally distributed across diagnostic groups and patients and controls were matched on education.

## 6. Future Directions

Future research should examine in more detail which cognitive abilities are linked to specific arithmetic errors. This preliminary study examined a topic that has been neglected to date through the comparison of demographically similar groups of participants, thus allowing for the control of possible influencing factors (e.g., age, sex, educational level, socioeconomic status). One point for future research studies is the inclusion and simultaneous assessment of additional relevant variables to detect whether they can predict cognitive performance (and more specifically arithmetic errors). Future interventions could incorporate such findings and greatly benefit from the knowledge of the exact types of arithmetic errors that occur in patients suffering from different neurocognitive disorders. The understanding of different levels of severity of neurocognitive disorders and their relationship to arithmetic errors, based on data coming from large samples, could assist differential diagnosis and prevent elder financial abuse [29].

## Figures and Tables

**Table 1:** General demographic and clinical information about the sample.

Demographics	Alzheimer’s Disease (AD) *N* = 110	Mild Cognitive Impairment (MCI) *N* = 107	Parkinson’s Disease Dementia (PDD) *N* = 94	Healthy Controls *N* = 109
Biological sex (*n* = female)	50	61	57	60
Age (in years)	75.20 (6.57)	73.53 (6.51)	72.73 (5.48)	74.18 (7.69)
Education (in years)	8.11 (3.71)	9.28 (3.98)	8.14 (3.41)	9.08 (4.88)
MMSE	17.20 (4.89)	27.11 (2.10)	22.67 (3.39)	29.51 (0.67)
GDS-15	3.90 (4.56)	2.29 (3.17)	3.10 (1.07)	2.24 (3.00)

**Table 2:** How many 5-cent coins make up EUR 1? Responses according to diagnosis.

Responses	Diagnoses				Total
	Alzheimer’s Disease	Mild Cognitive Impairment	Healthy Controls	Parkinson’s Disease Dementia	
Correct response	9	16	80	0	105
Adjusted residual	−4.7	−2.8	13.6	−6.4	
Place value errors	14	17	4	70	105
Adjusted residual	−3.5	−2.5	−6.0	12.6	
Magnitude errors	17	24	3	21	65
Adjusted residual	0.0	2.3	−4.3	2.1	
Procedural errors	26	29	11	3	69
Adjusted residual	2.4	3.5	−2.1	−3.9	
Other errors	44	21	11	0	76
Adjusted residual	6.9	0.5	−2.5	−5.2	
Total	110	107	109	94	420

**Table 3:** How many 50-cent coins make up EUR 15.50? Responses according to diagnosis.

Responses	Diagnoses				Total
	Alzheimer’s Disease	Mild Cognitive Impairment	Healthy Controls	Parkinson’s Disease Dementia	
Correct response	9	16	80	0	105
Adjusted residual	−4.7	−2.8	13.6	6.4	
Place value errors	25	21	8	14	68
Adjusted residual	2.2	1.1	−2.9	−0.4	
Magnitude errors	23	19	7	48	97
Adjusted residual	−0.6	−1.5	−4.8	7.3	
Procedural errors	29	24	8	15	76
Adjusted residual	2.6	1.3	−3.4	−0.6	
Other errors	24	27	6	17	74
Adjusted residual	1.3	2.4	−3.9	0.1	
Total	110	107	109	94	420

## Data Availability

The data presented in this study are available on request from the corresponding author.
